# Combined open revascularization and endovascular treatment of complex intracranial aneurysms: case series

**DOI:** 10.3389/fneur.2023.1102496

**Published:** 2023-04-21

**Authors:** Robert C. Rennert, Vincent N. Nguyen, Aidin Abedi, Nadia A. Atai, Joseph N. Carey, Matthew Tenser, Arun Amar, William J. Mack, Jonathan J. Russin

**Affiliations:** ^1^Department of Neurological Surgery, Keck School of Medicine, University of Southern California, Los Angeles, CA, United States; ^2^Neurorestoration Center, Keck School of Medicine, University of Southern California, Los Angeles, CA, United States; ^3^Division of Plastic and Reconstructive Surgery, Department of Surgery, Keck School of Medicine, University of Southern California, Los Angeles, CA, United States

**Keywords:** cerebral revascularization, bypass, aneurysm, neuroendovascular approach, embolization

## Abstract

**Background and purpose:**

The treatment of complex intracranial aneurysms can be challenging with stand-alone open or endovascular techniques, particularly after rupture. A combined open and endovascular strategy can potentially limit the risk of extensive dissections with open-only techniques, and allow for aggressive definitive endovascular treatments with minimized downstream ischemic risk.

**Materials and methods:**

Retrospective, single-institution review of consecutive patients undergoing combined open revascularization and endovascular embolization/occlusion for complex intracranial aneurysms from 1/2016 to 6/2022.

**Results:**

Ten patients (4 male [40%]; mean age 51.9 ± 8.7 years) underwent combined open revascularization and endovascular treatment of intracranial aneurysms. The majority of aneurysms, 9/10 (90%), were ruptured and 8/10 (80%) were fusiform in morphology. Aneurysms of the posterior circulation represented 8/10 (80%) of the cases (vertebral artery [VA] involving the posterior inferior cerebellar artery [PICA] origin, proximal PICA or anterior inferior cerebellar artery/PICA complex, or proximal posterior cerebral artery). Revascularization strategies included intracranial-to-intracranial (IC-IC; 7/10 [70%]) and extracranial-to-intracranial (EC-IC; 3/10 [30%]) constructs, with 100% postoperative patency. Initial endovascular procedures (consisting of aneurysm/vessel sacrifice in 9/10 patients) were performed early after surgery (0.7 ± 1.5 days). In one patient, secondary endovascular vessel sacrifice was performed after an initial sub-occlusive embolization. Treatment related strokes were diagnosed in 3/10 patients (30%), largely from involved or nearby perforators. All bypasses with follow-up were patent (median 14.0, range 4–72 months). Good outcomes (defined as a Glasgow Outcomes Scale ≥4 and modified Rankin Scale ≤2) occurred in 6/10 patients (60%).

**Conclusion:**

A variety of complex aneurysms not amenable to stand-alone open or endovascular techniques can be successfully treated with combined open and endovascular approaches. Recognition and preservation of perforators is critical to treatment success.

## Introduction

Treatment of complex, non-saccular intracranial aneurysms has increasingly involved endovascular therapies, in particular flow diversion, due to an ability to remodel diseased vessels from the inside in a minimally invasive manner (1). Intrasaccular devices similarly have the potential to change treatment paradigms for selected wide-necked saccular aneurysms (2). Limitations to these technologies nonetheless exist, particularly in the setting of ruptured aneurysms where flow diversion (or stent/coil constructs) requires the use of dual anti-platelet agents (increasing the risk of hemorrhage with ventricular catheters), and both flow diversion and intrasaccular devices can often have continued aneurysm filling (maintaining a small but significant risk of re-rupture) (3, 4). Although newer flow diversion technologies are being investigated that require only a single anti-platelet agent, concerns regarding significant morbidity and mortality from ischemic complications or post-treatment aneurysmal re-bleeding (occurring in up to 21% of patients) remain based on data from preliminary studies (5).

Open management of complex aneurysms, in particular cerebral bypass, remains an important tool for vascular neurosurgeons (6). Combined approaches that leverage the respective advantages of open and endovascular treatments, while avoiding their risks, can also be tailored to optimize the treatment of select aneurysms. For example, for fusiform or complex saccular ruptured aneurysms where a definitive treatment is desired, a combined strategy of open revascularization followed by endovascular deconstruction can be used to limit the risk of additional dissection with open-only techniques (i.e., for clip trapping), and allow for endovascular vessel sacrifice (or aggressive aneurysm treatment that may risk parent vessel occlusion) without downstream ischemic risk. We herein report our experience with combined open bypass and endovascular management of complex cerebral aneurysms.

## Materials and methods

This study was performed in compliance with Health Insurance Portability and Accountability Act and local institutional review board (IRB) regulations. Patient consent was obtained based on institutional guidelines.

Data from all patients undergoing combined open revascularization and endovascular treatment for the management of intracranial aneurysms from 1/2016 to 6/2022 was retrospectively collected from an IRB-approved, prospectively maintained database. Recorded data included: patient demographics (age, gender), presenting symptoms, clinical status, Glasgow Coma Scale (GCS) score, aneurysm characteristics, surgical and endovascular details, treatment/overall complications, and neurologic outcomes (Glasgow outcomes scale [GOS] and modified Rankin Scale [mRS] scores at discharge and last follow up). Descriptive statistics were used to report continuous data (mean ± standard deviation).

Management and planning of combined open revascularization and endovascular treatment of all patients in this series was based on multidisciplinary (open and endovascular) neurosurgical review of the patient, and neurovascular and pathologic anatomy. Surgical details for open revascularization strategies are described previously (7–11). All patients were maintained on aspirin in the peri-operative and post-operative period to promote bypass patency. Secondary endovascular procedures were performed as early as possible after revascularization to secure the aneurysm (in cases of rupture) and to promote bypass patency. Systemic heparinization was not used during endovascular procedures for ruptured aneurysms or for procedures immediately following open surgery. Neuromonitoring was used for both open and endovascular procedures. Balloon test occlusion (BTO) was performed if feasible based on vascular anatomy to aid in treatment planning prior to endovascular vessel sacrifice.

## Results

**Figure 1:**
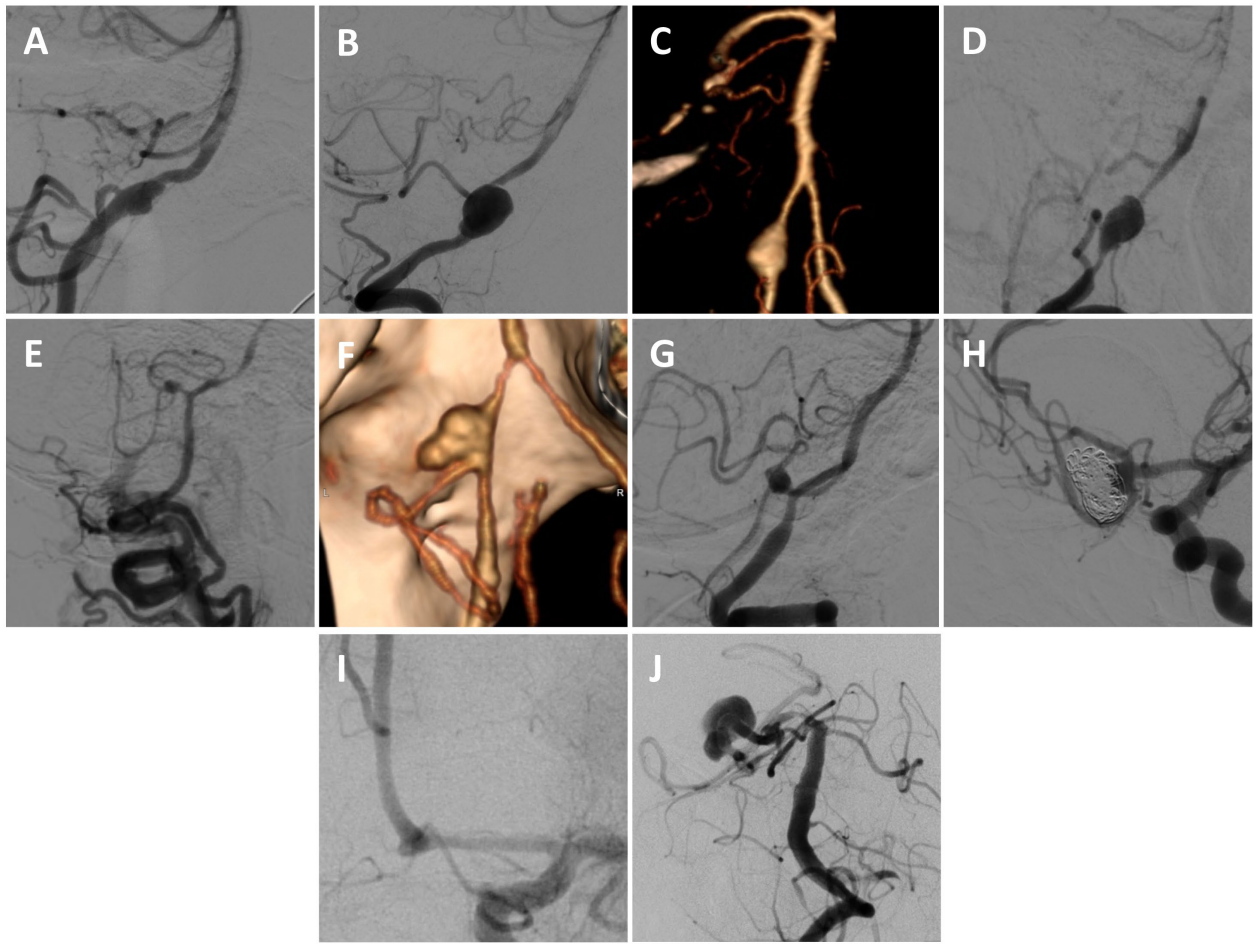
Treated aneurysms. **(A)** Left VA fusiform ruptured aneurysm involving the proximal left PICA (lateral angiogram of left VA). **(B)** Right VA fusiform ruptured aneurysm involving the proximal right PICA (lateral angiogram of the right VA). **(C)** Left VA fusiform ruptured aneurysm involving the proximal left PICA (3D-reconstruction). **(D)** Right VA fusiform ruptured aneurysm involving the proximal right PICA (lateral angiogram of the right VA). **(E)** Right proximal AICA/PICA fusiform ruptured aneurysm involving the vessel origin (lateral angiogram of the right VA, also demonstrating an iatrogenic right cervical VA-venous fistula from an unsuccessful prior endovascular treatment attempt). **(F)** Left VA fusiform ruptured aneurysm involving the proximal left PICA (3D-reconstruction). **(G)** Left proximal PICA fusiform ruptured aneurysm (lateral angiogram of left VA). **(H)** Recurrent ruptured and partially coiled Acomm aneurysm arising from an azygous left A1 (oblique angiogram of the left ICA). **(I)** Left A1-2 junction traumatic ruptured pseudoaneurysm (oblique angiogram of the left ICA). **(J)** Large right fusiform P2 aneurysm with a proximal dysplastic P1 (AP angiogram of the left VA).

Ten patients (4 male [40%]; mean age 51.9 ± 8.7 years) underwent combined endovascular and open treatment of intracranial aneurysms (Figure 1; Table 1). Most aneurysms presented ruptured, 9/10 (90%), and 3/10 (30%) patients had undergone prior aneurysm treatments or treatment attempts. Aneurysm locations included: 5 (50%) involving the vertebral artery (VA)/posterior inferior cerebellar artery (PICA) origin, 2 (20%) of the proximal PICA or anterior inferior cerebellar artery (AICA)/PICA complex, 2 (20%) of the anterior communicating artery (Acomm) or A1/2 junction, and 1 (10%) of the proximal posterior cerebral artery (PCA). All (8/8) of the aneurysms involving the posterior circulation were fusiform, while aneurysms involving the anterior circulation were traumatic/pseudoaneurysm (1/2; 50%) or saccular (1/2; 50%) in morphology. Bypass strategies included both extracranial-to-intracranial (EC-IC) and intracranial-to-intracranial (IC-IC) constructs and included PICA-PICA side-to-side (5/10; 50%), V3-PICA with a descending branch of the lateral circumflex artery (DLCFA) interposition graft (2/10; 20%), A3-A3 side-to-side (2/10; 20%), and occipital artery (OA)-P4 with a DLCFA interposition graft (1/10; 10%). Mean temporary clip time was 38.5 ± 8.9 min, and immediate bypass patency was 100%. Initial endovascular procedures were performed early after surgery (mean 0.7 ± 1.5 days) to reduce rupture risk and promote bypass patency, and included vessel sacrifice in 9/10 patients (90%) (Table 2). Two patients underwent multiple endovascular embolizations. In one patient, a non-occlusive post-bypass endovascular strategy was initially employed for a fusiform VA aneurysm involving the proximal left PICA to preserve flow to the dominant anterior spinal artery (ASA) located immediately distal to the aneurysm, although further aneurysmal growth necessitated a delayed vessel sacrifice. The second patient had an initial post-bypass endovascular strategy of aggressive re-coiling of a recurrent/re-ruptured Acomm aneurysm arising from an unpaired A1 (including Acomm sacrifice), followed by delayed flow diversion/coiling for aneurysm re-growth.

**Table 1:** Patient summary.

**Patient #**	**Age/Sex**	**Presenting symptoms**	**Ruptured (Y/N)**	**Initial SAH grade**	**Initial GCS**	**Stroke at presentation**	**Previous aneurysm treatment**	**Aneurysm**	**Revascularization procedure**	**Temporary clip time (minutes)**	**Anastomosis patency**	**Endovascular procedure**	**Treatment-related stroke**	**Other notable events**	**GOS at D/c**	**mRS at D/c**	**F/u (months)**	**Anastamosis patency on F/u**	**GOS at F/u**	**mRS at F/u**
**1**	41/F	Severe HA -> LOC	Y	HH4F4	9	Chronic right superior frontal gyrus stroke	N	Left VA fusiform aneurysm involving the proximal left PICA	PICA-PICA bypass	38	Y	Coil embolization/vessel sacrifice of left VA aneurysm	N	N	5	0	60	Y	5	0
**2**	41/M	HA, blurry vision	Y	HH1F2	15	N	N	Right VA fusiform aneurysm involving the proximal right PICA	PICA-PICA bypass	40	Y	Coil embolization/vessel sacrifice of right VA aneurysm	N	N	5	0	n/a	n/a	n/a	n/a
**3**	44/M	Severe HA, nausea	Y	HH1F3	15 - re-rupture upon transfer from OSH requiring intubation	N	N	Left VA fusiform aneurysm involving the proximal left PICA	Left V3 to left tonsillar PICA bypass w/ DLCFA interposition graft	30	Y	Coil embolization of left VA aneurysm; secondary coil/liquid embolic embolization/sacrifice of left VA aneurysm after expansion	ASA stroke following VA artery sacrifice	N	3	5	n/a	n/a	n/a	n/a
**4**	48/F	HA -> seizure	Y	HH3F3	13	N	N	Right VA fusiform aneurysm involving the proximal right PICA	PICA-PICA bypass	50	Y	Coil/liquid embolic embolization/vessel sacrifice of right VA aneurysm	N	Severe vasospasm - delayed right lateral medullary stroke	3	3	72	Y	4	3
**5**	66/F	HA, nausea -> AMS	Y	HH4F4	10	Right cerebellar strokes	Previous unsuccessful coil attempt; iatrogenic right cervical VA-venous fistula s/p stenting	Right proximal AICA/PICA fusiform aneurysm involving the vessel origin	PICA-PICA bypass	40	Y	Coil embolization/vessel sacrifice of right AICA/PICA aneurysm; coil/liquid embolic embolization/sacrifice of right VA at level of C2 fistula	N	N	3	4	19	Y	5	0
**6**	57/F	LOC	Y	HH4F4	8	N	N	Left VA fusiform aneurysm involving the proximal left PICA	PICA-PICA bypass	28	Y	Coil/liquid embolic embolization/vessel sacrifice of left VA aneurysm	Left lateral medullary stroke following VA artery sacrifice	N	3	5	n/a	n/a	n/a	n/a
**7**	59/F	HA -> seizure	Y	HH3F4	8	N	N	Left proximal PICA fusiform aneurysm	Right V3 to left tonsillar PICA bypass w/ DLCFA interposition graft	51	Y	Coil embolization/vessel sacrifice of left PICA aneurysm	N	N	4	2	4	Y	4	2
**8**	61/M	HA -> AMS	Y	HH2F3	13	N	Coiling after initial rupture, re-coiling after growth and re-rupture with significant residual aneurysm	Large, multilobed saccular Acomm aneurysm arising from an unpaired left A1	A3-A3 bypass	48	Y	Coil embolization of residual Acomm aneurysm with Acomm sacrifice; delayed flow diversion and coiling of recurrent aneurysm	N	N	5	1	8	Y	5	0
**9**	49/F	Iatrogenic injury during transsphenoidal resection of recurrent pituitary tumor	Y	HH4F4	6T	Left fronto-basal	N	Left A1-2 junction pseudoaneurysm	A3-A3 bypass	32	Y	Coil embolization/vessel sacrifice of pseudoaneurysm and left A1-2 junction	N	Severe vasospasm - delayed bilateral ACA strokes; required decompressive hemicraniectomy	3	5	n/a	n/a	n/a	n/a
**10**	53/M	Indidental	N	n/a	15	N	Aborted flow diversion endovascular treatment	Large right fusiform P2 aneurysm with proximal dysplastic vessel	Right OA to P4 bypass w/ DLCFA interposition graft	28	Y	Coil embolization/vessel sacrifice of right P2 aneurysm	Small right thalamic and occipital strokes following PCA sacrifice	N	4	2	9	Y	4	2

A1, first segment of the anterior cerebral artery; A2, second segment of the anterior cerebral artery; A3, third segment of the anterior cerebral artery; ACA, anterior cerebral artery; Acomm, anterior communication artery; AICA, anterior inferior cerebellar artery; AMS, altered mental status; ASA, anterior spinal artery; DLCFA, descending branch of the lateral circumflex artery; F, female; GCS, Glasgow coma score; HA, headache; HH/F, Hunt & Hess and Fisher score; LOC, loss of consciousness; M, male; N, no; n/a, not applicable; OA, occipital artery; P2, second segment of the posterior cerebral artery; P4, fourth segment of the posterior cerebral artery; PCA, posterior cerebral artery; PICA, posterior inferior cerebellar artery; V3, third segment of the vertebral artery; VA, vertebral artery; Y, yes.

**Table 2:** Summary of treatment details.

Patient #	Aneurysm	Bypass	Endovascular procedure	Days after open bypass	Aneurysm dimensions (mm)	BTO	Balloon-assisted intervention	Guide/intermediate catheters (in)	Microcatheter/wire (in)	Implants	Liquid embolic	Intraprocedural heparinization	Antiplatelets
1	VA/proximal PICA	PICA-PICA	Embolization/sacrifice	0	10 × 6 × 5	N	Y - extra-compliant balloon	0.070 ID delivery (VA)	0.017 ID microcatheter; 0.014 microwire	Balloon catheter: 5 coils, 6–3 mm diameter; Microcatheter: 9 coils, 4–2 mm diameter	Y - Non-adhesive liquid embolic	N	Aspirin
2	VA/proximal PICA	PICA-PICA	Embolization/sacrifice	0	11 × 10 × 9	N	N	0.087 ID guide (subclavian); 0.054 ID intermediate (VA)	0.017 ID microcatheter; 0.014 microwire	19 coils, 10–1 mm diameter	N	N	Aspirin
3	VA/proximal PICA	V3-DLCFA-PICA	Embolization	1	7 × 6 × 5	N	N	0.087 ID guide (subclavian); 0.055 ID intermediate (VA)	0.017 ID microcatheter; 0.012 microwire	5 coils, 6–2 mm diameter	N	N	Aspirin
3			Embolization/sacrifice	9	9 × 7 × 5 - filling around coil mass	Y - extra-compliant balloon	Y - extra-compliant balloon	0.087 ID guide (subclavian); 0.055 ID intermediate (VA)	0.017 ID microcatheter; 0.012 microwire	8 coils, 4–1.5 mm diameter	Y - Non-adhesive liquid embolic	Partial (2000 U) to balance risk of thrombosis and enlarging aneurysm	Aspirin
4	VA/proximal PICA	PICA-PICA	Embolization/sacrifice	0	9 × 5 × 5	N	N	0.087 ID guide (subclavian); 0.055 ID intermediate (VA)	0.017 ID microcatheter; 0.014 microwire	11 colils, 7–2 mm diameter	Y - Non-adhesive liquid embolic	N	Aspirin
5	Proximal AICA/PICA	PICA-PICA	Embolization/sacrifice of aneurysm; Embolization/sacrifice of proximal VA fistula	0	5 × 3 × 3	N	N	0.087 ID guide (subclavian); 0.055 ID intermediate (VA)	0.016 ID microcatheter; 0.014 microwire	AICA/PICA: 7 coils, 3–1 mm diameter; VA fistula: 21 coils, 3.5–2 mm diameter	Y - Non-adhesive liquid embolic	N	Aspirin
6	VA/proximal PICA	PICA-PICA	Embolization/sacrifice	1	8 × 4 × 3	N	Y - extra-compliant balloon	0.087 ID guide (subclavian); 0.070 ID intermediate (VA)	0.017 ID microcatheter; 0.014 microwire	Microcatheter: 3 coils, 6–4 mm diameter; Balloon catheter: 5 coils, 4–2 mm diameter	Y - Non-adhesive liquid embolic	N	Aspirin
7	Proximal PICA	V3-DLCFA-PICA	Embolization/sacrifice	0	5 × 3 × 3	N	N	0.087 ID guide (subclavian); 0.055 ID intermediate (VA)	0.016 ID microcatheter; 0.014 microwire	12 coils, 4–1.5 mm diameter	N	N	Aspirin
8	Acomm from an unpaired A1	A3-A3	Embolization/Acomm sacrifice	5	Originally 12 × 11 × 11; 9 × 4 × 3 at time of bypass/embolization	N	N	0.087 ID guide (proximal ICA); 0.055 ID intermediate (distal ICA)	0.017 ID and 0.016 ID microcatheters; 0.014 microwire	13 coils, 5–1.5 mm diameter	N	N	Aspirin
8			Flow diversion/embolization	45	7 × 4 × 3 at time of flow diversion/embolization	Attempted but unable to track compliant balloon to A1/2 junction	N	0.10 ID guide (CCA); 0.044 ID intermediate (distal ICA)	Flow diversion: 0.022 ID microcatheter; 0.014 microwire; Embolization: 0.017 ID microcatheter; 0.014 microwire	Flow diverter, 3.5 × 16 mm; 21 coils, 6–2 mm diameter	N	Y	Aspirin, Prasugrel
9	A1-2 pseudoaneurysm	A3-A3	Embolization/sacrifice	0	3 × 2 × 2	N	N	0.088 ID delivery (ICA)	0.017 ID microcatheter; 0.014 microwire	5 coils, 3–2.5 mm diameter	N	N	Aspirin
10	P2	OA-DLCFA-P4	Embolization/sacrifice	0	16 × 12 × 11	Y - mini compliant balloon	N	0.087 ID guide (subclavian); 0.055 ID intermediate (VA)	0.017 ID microcatheter; 0.014 microwire	17 coils, 12–1.5 mm diameter	N	N	Aspirin

A1, first segment of the anterior cerebral artery; A2, second segment of the anterior cerebral artery; A3, third segment of the anterior cerebral artery; Acomm, anterior communication artery; AICA, anterior inferior cerebellar artery; BTO, balloon test occlusion; DLCFA, descending branch of the lateral circumflex artery; ID, inner diameter; N, no; n/a, not applicable; OA, occipital artery; P2, second segment of the posterior cerebral artery; P4, fourth segment of the posterior cerebral artery; PICA, posterior inferior cerebellar artery; V3, third segment of the vertebral artery; VA, vertebral artery; Y, yes.

Strokes from any etiology occurred in 5/10 patients (50%). Treatment related strokes were diagnosed in 3/10 patients (30%) and included a lateral medullary stroke after PICA-PICA bypass and VA sacrifice, thalamic/occipital strokes after PCA bypass/sacrifice, and an ASA stroke after V3-PICA bypass and delayed VA sacrifice. In 2/10 patients (20%) strokes were from vasospasm. Mean GOS and mRS at discharge were 3.8 ± 0.9 and 2.7 ± 2.0, respectively. Follow up data was available in 6/10 patients (60%). Over a median follow up of 14.0 months (range 4–72), all bypasses were patent and mean GOS and mRS were 4.5 ± 0.5 and 1.2 ± 1.3, respectively. Good outcomes (defined as a GOS ≥ 4 and mRS ≤ 2) occurred in 6/10 patients (60%) overall.

### Case examples

#### Patient #7

**Figure 2:**
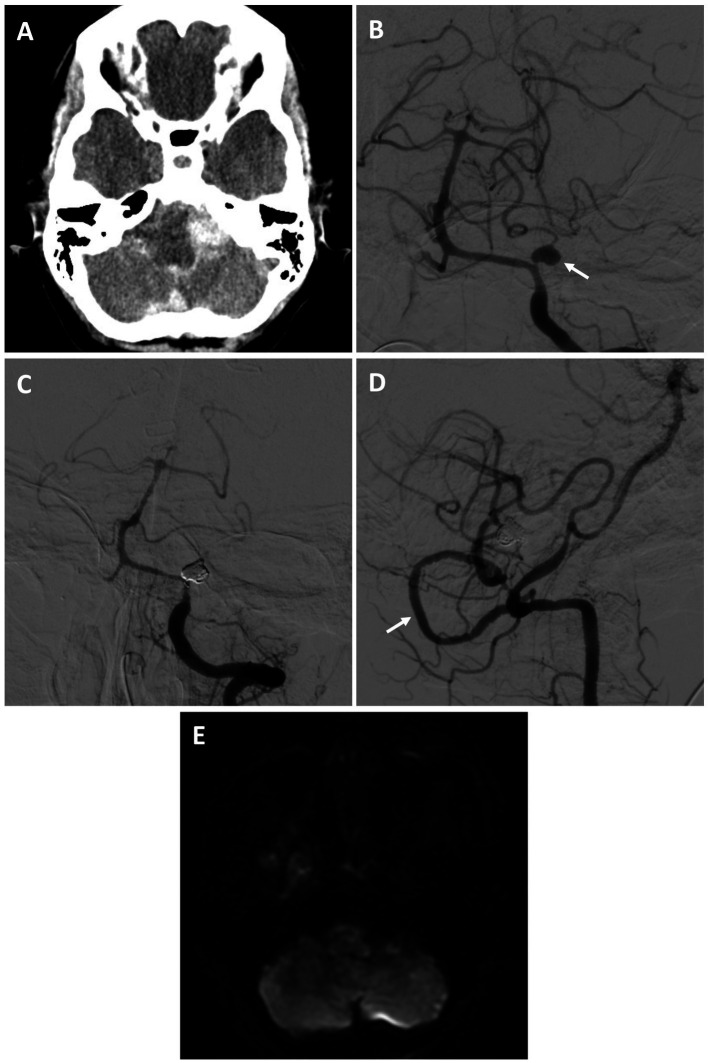
Patient #7. A 59-year-old female experienced a Hunt and Hess 3, Fisher 4 SAH from a left proximal PICA fusiform aneurysm [**(A)** axial non-contrast head CT; **(B)** AP angiogram of the left vertebral artery (arrow highlighting aneurysm)]. She underwent a V3 to PICA bypass using a DLCFA interposition graft, followed by internal PICA aneurysm embolization. She had no strokes and an uneventful recovery. On 4-month follow was performing all activities of daily living independently. Angiogram demonstrating PICA occlusion [**(C)** AP left vertebral artery injection, and patent bypass **(D)** lateral right vertebral artery injection (arrow highlights bypass)]. **(E)** Post-procedural MRI (axial DWI) demonstrated no strokes.

A 59-year-old female who was recovering at an outside hospital from a perforated diverticulum experienced a severe acute headache followed by a seizure and was found to have diffuse subarachnoid hemorrhage (SAH) and obstructive hydrocephalus from a ruptured left proximal PICA fusiform aneurysm (Hunt and Hess 3, Fisher 4) (Figures 2A,B). She was stabilized with an external ventricular drain and transferred to our facility. She was recommended for a combined open revascularization and endovascular aneurysm/vessel occlusion on multidisciplinary review. As the contralateral PICA was not favorable for IC-IC bypass a V3 to left tonsillar PICA bypass using a DLCFA interposition graft was performed after a suboccipital craniotomy and C1 laminectomy. Immediately afterwards, the left PICA aneurysmal segment was internally embolized with coils (Figures 2C,D). She had an uneventful hospital course without complications (Figure 2E). On four-month follow up she was performing all activities of daily living independently.

#### Patient #8

**Figure 3:**
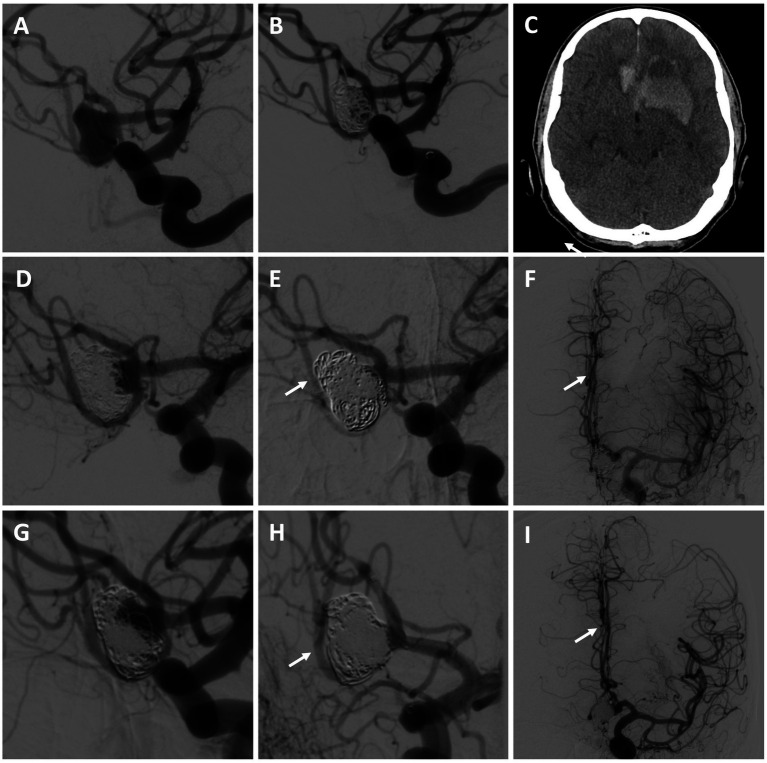
Patient #8. A 61-year-old male presented with SAH and intraparenchymal hemorrhage from a previously embolized Acomm aneurysm arising from an unpaired left A1 [**(A)** left ICA angiogram demonstrating initial Acomm aneurysm, and after coil embolization **(B)**; **(C)** head CT demonstrating SAH and IPH]. A repeat coil embolization was performed, leaving a neck residual to preserve the Acomm [**(D)** post re-coiling angiogram demonstrating a neck residual and patent Acomm]. An A3-A3 bypass was performed, followed by repeat aneurysm embolization with Acomm sacrifice [**(E,F)** post-treatment angiogram demonstrating no residual aneurysm and filling of the right ACA through the bypass (highlighted by arrows)]. **(G)** 3-month repeat angiogram demonstrated aneurysm re-growth and an open Acomm, treated with flow diversion across the left A1-2 and repeat aneurysm coiling (including Acomm re-sacrifice) [**(H,I)** post-treatment angiogram demonstrating no residual aneurysm and filling of the right ACA through the bypass (highlighted by arrows)]. On follow up, the bypass was open and there was no aneurysm recurrence. The patient was neurologically intact and living independently.

A 61-year-old male with a history of coil embolization of a ruptured (Hunt and Hess 1, Fisher 2), large, multilobed Acomm aneurysm arising from an unpaired left A1 presented 3 months later with SAH (Hunt and Hess 2, Fisher 3) and intraparenchymal hemorrhage, with aneurysm re-growth (Figures 3A–C). A repeat coil embolization was performed, leaving a neck residual to preserve the Acomm (Figure 3D). He was recommended for a right anterior cerebral artery (ACA) revascularization to allow aggressive Acomm aneurysm coiling and possible Acomm sacrifice given the rapid re-growth of the lesion. He underwent an A3-A3 bypass followed by aneurysm embolization with Acomm sacrifice 5 days later (as the aneurysm dome was already secure), allowing for filling of the right ACA through the bypass (Figures 3E,F). He had an uneventful post-operative course and was discharged home without focal neurologic deficits. On 3-month repeat angiogram the aneurysm had re-grown and the Acomm was patent (Figure 3G). A BTO was attempted to assess the bypass, however, the balloon was unable to be positioned. Flow diversion across the left A1-2 and repeat aneurysm coiling (including re-sacrifice of the Acomm) was performed (Figures 3H,I). After an uneventful recovery, he was discharged home neurologically intact. On 4-month follow-up angiogram there was no residual aneurysm, and the bypass was patent.

#### Patient #10

**Figure 4:**
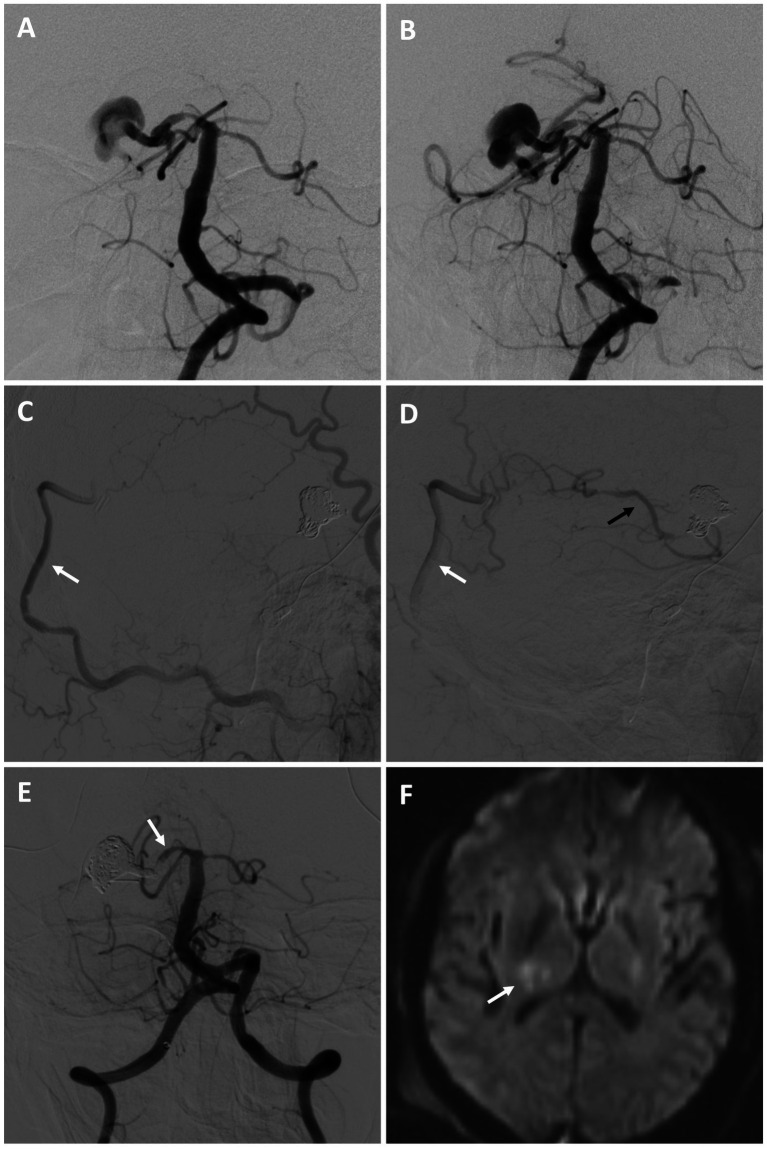
Patient #10. A 53-year-old male was referred for a large right fusiform P2 aneurysm with a proximal dysplastic P1 vessel [**(A,B)** AP angiogram of the left vertebral artery]. A prior attempt at flow diversion was aborted due to unfavorable anatomy. He underwent a right OA to P4 bypass with a DLCFA interposition graft, followed by a coil embolization/vessel sacrifice of the right P2 aneurysm (keeping the proximal right P1 patent) after BTO [**(C,D)** serial lateral images from a right external carotid artery injection demonstrating patency of the bypass (white arrows) and backfilling of the PCA territory (black arrow)]. The right P1 was kept open to preserve perforator flow [**(E)** AP angiogram of the right vertebral artery (arrow highlights right P1)]. Post-operatively he experienced a small right thalamic stroke [**(F)** axial diffusion-weighted MRI, arrow highlighting stroke], likely from perforators off the severely diseased and sacrificed P2 segment, as well as a small right occipital stroke of unclear etiology (not shown). He had left mild hemiparesis and a partial left hemianopsia. This significantly improved on 9-month follow, where he was living at home and performing all activities of daily living independently.

A 53-year-old male with a history of chronic kidney disease was referred for a large right fusiform P2 aneurysm with a proximal dysplastic P1 vessel (Figures 4A,B) after an unsuccessful attempt at flow diversion at an outside facility. He was neurologically intact. He was recommended for a combined open revascularization and endovascular internal embolization on multidisciplinary review, with a plan to keep the proximal P1 open, despite its dysplastic appearance, to preserve perforator flow. He underwent an uneventful right OA to P4 bypass with a DLCFA interposition graft. The same day he underwent a right P1 BTO that confirmed bypass patency and did not result in neurologic changes. This was followed by a coil embolization/vessel sacrifice of the right P2 aneurysm (Figures 4C–E). Flow into the proximal right P1 was preserved. He experienced a small right thalamic stroke following aneurysm sacrifice (Figure 4F), likely from perforators off the severely diseased and sacrificed P2 segment, as well as a small right occipital stroke. This resulted in mild left hemiparesis and a partial left hemianopsia that required a short inpatient rehabilitation stay. On 9-month follow up he was living at home and performing all activities of daily living independently.

## Discussion

Combining open and endovascular techniques has established utility for a variety of cerebrovascular pathologies, including complex arteriovenous malformations, arteriovenous fistulas, and aneurysms (12–22). Such approaches are especially useful for aneurysms that can be challenging to treat with purely open or endovascular options, including ruptured giant, blister, fusiform, dissecting, heavily calcified, or pseudoaneurysms in difficult to access locations and with associated perforating vessels or scar tissues from prior treatments (13). Described strategies include both endovascular therapy after surgical treatment (i.e., coil embolization after partial clipping of incompletely exposed or recurrent aneurysms, or endovascular occlusion/vessel sacrifice after bypass, as used herein), and open surgical treatment after endovascular therapy (i.e., definitive clipping after dome-protection of ruptured aneurysms unable to be completely coiled, or surgical debulking to reduce mass effect after endovascular occlusion) (12, 13, 15).

Our data add to prior works supporting the use of cerebral bypass combined with endovascular embolization for complex intracranial aneurysms (13, 14, 16, 17, 19, 21, 23–26). Although previously described for lesions throughout the anterior and posterior circulation, we have found these constructs most useful for the treatment of aneurysms less surgically accessible, particularly in the posterior circulation such as those located on the distal VA, proximal PICA, and proximal PCA (80% of cases in this series). In our experience, complex aneurysms of the anterior circulation (internal carotid artery [ICA], middle cerebral artery [MCA], and even ACA) can often be directly addressed with open surgery algorithms due to their more easily accessible anatomy, which facilitates direct visualization of perforators for precise clip application during vessel ligation, trapping or aneurysm/vessel reconstruction (8, 27–31). This is not always feasible or advisable though, as evidenced by the two ACA cases in this series where a combined open and endovascular approach was used to avoid manipulation of an unstable A1/A2 pseudoaneurysm and a multiply ruptured partially coiled wide-necked Acomm aneurysm.

As demonstrated in this series, the combined approach is easily tailored to a variety of aneurysms and anatomical variations. Emerging data supports the technical and safety profile of IC-IC versus EC-IC constructs (8, 9, 32–35), and we prefer to use PICA-PICA bypass for revascularization during treatment of complex proximal PICA or VA/PICA aneurysms when the contralateral PICA anatomy is favorable. This also avoids the need for dissection of the often torturous OA or harvest of an interposition graft (as needed with other EC-IC options), or performance of a deep anastomosis (as needed with excision and re-anastomosis/re-implantation strategies) (9, 33). In cases where the contralateral PICA is not favorable for side-to-side IC-IC bypass, the DLCFA is well-sized for PICA revascularization and can be used as an interposition graft with a V3, OA, or even the contralateral PICA as a donor vessel (10, 11). Regardless of the bypass strategy used, when combined with endovascular sacrifice of the proximal PICA (and VA as needed), extended skull base approaches to reach the distal VA/PICA origin anterolateral to the brainstem can be avoided. With this approach, after vessel sacrifice the distal PICA fills anterograde from its anastomosis at the tonsillar segment, while the proximal PICA (including brainstem perforators) fills in a retrograde fashion to the point of occlusion. A similar concept can be employed for complex proximal ACA aneurysms by combining an A3-A3 bypass with endovascular vessel sacrifice or aggressive aneurysm treatments that risk vessel occlusion. This strategy avoids the need for long interposition grafts to reach into the interhemispheric fissure from the external carotid artery (ECA) circulation for ACA revascularization, while also avoiding surgical manipulation of the perforator rich A1/A2 region for aneurysms with expected scarring or wall friability. For PCA revascularizations as part of combined treatments of complex proximal PCA aneurysms, IC-IC constructs are less favorable, and we prefer to use a DLCFA interposition graft to connect the OA to a P3 or P4 vessel (again avoiding a time-consuming full OA dissection) (7, 10, 11). This can be followed by proximal PCA endovascular sacrifice, avoiding temporal lobe retraction and potential venous compromise from an additional open subtemporal approach, as well as the manipulation of often sub-optimally visualized sensitive proximal PCA perforators.

Although the overall stroke rate was 50% in this series (5 patients), treatment-related strokes occurred in three patients (30%), with the remaining two patients (20%) having delayed strokes from vasospasm. With the use of multiple treatments in a combined approach, the additional risk of each procedure should be offset by its advantages. The 100% bypass patency and low open surgery complication rate herein is similar to the 97% patency reported in a recent large series of 430 cerebral bypasses (36). These data support the relative efficacy of bypass in experienced hands, and justify the addition of an open procedure to minimize the ischemic risk associated with stand-alone end-vessel sacrifice (37, 38). Vessel sacrifice by any means (open or endovascular) is nonetheless associated with the risk of both downstream and perforator strokes, with bypass mitigating the downstream risk but not the risk of perforator stroke if they arise directly from the diseased vessel and a trapping/occlusive (rather than proximal Hunterian ligation) strategy is used. In all the patients in this series, endovascular embolization was selected to avoid unfavorable open dissections, with any significant angiographically visualized perforators preserved and a minimum length of vessel sacrificed to secure the aneurysm.

Understanding perforator anatomy is crucial to designing a treatment strategy that minimizes stroke risk. Along the V4 VA, critical perforators (including the ASA) are variable but thought to be more common distal to the PICA origin (39–41). Accordingly, although brainstem strokes from perforator infarcts can occur from sacrifice anywhere along the V4 vessel, sacrifice of the segment distal to the PICA is thought to be higher risk than more proximally (39, 42). This is true even with selective sacrifice only at the level of disease, where although it is presumed that perforators within the pathologic segment have likely already been occluded, a stump effect from inadequate outflow can extend the length of vessel occlusion based on local arterial anatomy independent of sacrifice technique (42). Even with visualization and early angiographic preservation of perforators, their patency is thus not guaranteed with deconstructive options. This is demonstrated in the patient that experienced an ASA stroke in this series, which occurred after endovascular vessel sacrifice despite angiographic visualization of the ASA just distal to the aneurysm and allowing it to backfill from the contralateral VA. Learning from this case, we have since allowed ASA anatomy to dictate the treatment strategy of VA/PICA aneurysms, with Hunterian ligation or bypass/clip trapping used for ASAs arising from the aneurysm itself or just distal, respectively (allowing for direct, real-time assessments of ASA viability with clip placement and the potential for strategy modifications if changes are seen with vessel flow or neuromonitoring), and combined open bypass and endovascular occlusion used for cases where the ASA is removed from the aneurysm and the potential for a stump effect is low (43). Similarly, with PICA-PICA bypass, avoidance of tension on the PICAs is critical to maintain lateral medullary perforator patency, a potential contributing factor in the patient in this series with a lateral medullary stroke after PICA-PICA bypass and occlusion of a VA/PICA aneurysm. In this patient, mobilization of the ipsilateral PICA was needed to reach the contralateral vessel for bypass, potentially placing the proximal PICA perforators at risk and decreasing retrograde demand within this vessel. Learning from this, EC-IC PICA bypass has since been used if there is any concern for PICA tension with a PICA-PICA construct.

Perforators play a similarly important role in the treatment of proximal PCA aneurysms. Although these vessels can be difficult to visualize, the proximal PCA is perforator-rich and sacrifice should be avoided whenever possible (44). In the patient that experienced a small thalamic stroke from PCA perforators, this occurred despite keeping a portion of the diseased segment of the PCA proximal to the aneurysm open to allow for perforator filling (with the stroke likely from occlusion of a small vessel arising more distally along the sacrificed and grossly dysplastic P2). This situation is difficult to predict, and although it would likely have also occurred with clip trapping, may have been avoided with a bypass/proximal ligation strategy. Emerging technologies such as high-resolution cone beam computed tomography scans, which can visualize small vessels with high resolution, may help identify critical perforators pre-operatively and inform treatment planning focused on their preservation (45). BTOs may be helpful to assess bypass adequacy and predict tolerance of vessel occlusion prior to sacrifice (especially for vessels other than the VA with no expected redundancy, and/or to assess for potential stump affect with nearby critical perforators). It can nonetheless be challenging to position the balloon properly when sacrifice of smaller or more torturous vessels is planned (i.e., an ACA as in patient 8). BTOs are also not entirely predictive, as the BTO and final occlusion sites often differ slightly. Highlighting this point, patient 3 and patient 10 experienced an ASA and PCA perforator stroke, respectively, after unremarkable BTOs of the VA and P1 prior to aneurysm embolization/sacrifice.

With use of combined open and endovascular approaches in general, if there is concern for significant perforator occlusion with aneurysm embolization, proximal vessel occlusion or open clipping at a perforator-free area can be considered, allowing retrograde filling of the aneurysm and perforators through the bypass (i.e., Hunterian ligation). This must nonetheless be balanced by the reduced but ongoing rupture risk with continued aneurysm filling with this approach (46). Open clip trapping can also be used if a significant perforator is near but not emerging from the aneurysm and the proximal and distal vessel are surgically accessible.

An analysis of outcomes for the other main treatment options for complex intracranial aneurysms highlights the significant challenges of managing this patient population. Flow diversion is the main endovascular option for the treatment of nonsaccular ruptured aneurysms, but is associated with a 21 to 26% rate of ischemic/hemorrhagic complications with use of either standard devices and dual anti-platelet regimens, or newer devices and single agent anti-platelets across all anatomic sites (4, 5). While good neurologic outcomes have been reported with flow diversion for the treatment of fusiform VA aneurysms involving the PICA origin (representing 50% of the cases in the series), complete aneurysm occlusion occurs in <60% of cases, while PICA/VA occlusion can occur in up to 10% of cases (47). A variety of other endovascular options exist for complex non-saccular dissecting lesions, such as placement of multiple stents, stent-assisted coiling, and internal trapping with stenting (described as an alternative to flow diversion if a daughter vessel is involved), but are associated with 12.5 to 25% rates of infarction or aneurysm recurrence (48). Similarly, although selected complex saccular aneurysms not amenable to primary coiling and treatable with stenting or intrasaccular devices can have low rates of procedure-related hemorrhagic/thrombotic or external ventricular drainage–related events (<10% overall), persistent aneurysm filling with unclear long-term implications can occur in a significant proportion (17–46%) of cases. (2, 49). Nonetheless, the algorithm for aneurysms at our institution is endovascular management first, with more complex open or combined approaches reserved for lesions deemed to be poor candidates for stand-alone endovascular therapies (including flow diversion) on multidisciplinary review. While a direct comparison of open-only treatment strategies for the heterogenous complex aneurysm mix in this series is challenging, data from a recent series of 42 patients with largely ruptured dissecting aneurysms of the vertebrobasilar system (the majority of patients in our series) reflects the often poor baseline neurologic conditions of these patients, with good outcomes reported in less than 50% of patients with a variety of open treatments (50). The 60% overall rate of good outcomes in our series (despite 90% of patients having ruptured aneurysms), is consistent with prior data demonstrating the utility of a combined endovascular and open approach for otherwise difficult-to-treat aneurysms (16, 17).

Limitations of this study include its single-institution, retrospective design, and small patient size reflective of the relative rarity of complex aneurysms requiring combined treatments. Four patients (40%) were also lost to follow up, a reflection of the quaternary referral nature of our institution where transfer back to the referring center is often required after completion of the acute treatment stage. Finally, as expertise in both complex open revascularization and endovascular techniques is critical for the successful implementation of this strategy, the generalizability of this data is most pertinent to other high-volume bypass centers.

## Conclusion

Combined open revascularization and endovascular embolization can be used for select complex aneurysms not amenable to stand-alone open or endovascular techniques.

## Data availability statement

The original contributions presented in the study are included in the article/supplementary material, further inquiries can be directed to the corresponding author.

## Ethics statement

The studies involving human participants were reviewed and approved by University of Southern California Institutional Review Board. Written informed consent for participation was not required for this study in accordance with the national legislation and the institutional requirements.

## Author contributions

RR and JR contributed to the design and conception of the study. RR, VN, JR, JC, MT, AAm, and WM developed plans for and participated in the clinical care of included patients. AAb and NA organized and maintained the database. RR, VN, AAb, and NA collected data. RR drafted the manuscript. All authors contributed to the article and approved the submitted version.

## Conflict of interest

The authors declare that the research was conducted in the absence of any commercial or financial relationships that could be construed as a potential conflict of interest.

## Publisher’s note

All claims expressed in this article are solely those of the authors and do not necessarily represent those of their affiliated organizations, or those of the publisher, the editors and the reviewers. Any product that may be evaluated in this article, or claim that may be made by its manufacturer, is not guaranteed or endorsed by the publisher.
